# Draft Genome Sequence of *Arcobacter cibarius* Strain LMG21996^T^, Isolated from Broiler Carcasses

**DOI:** 10.1128/genomeA.00034-14

**Published:** 2014-02-20

**Authors:** Zaky Adam, Kerri Whiteduck-Leveillee, Michel Cloutier, Wen Chen, Christopher T. Lewis, C. André Lévesque, Edward Topp, David R. Lapen, James T. Tambong, Guylaine Talbot, Izhar U. H. Khan

**Affiliations:** a Eastern Cereal and Oilseed Research Centre (ECORC), Agriculture and Agri-Food Canada, Ottawa, Ontario, Canada; b Southern Crop Protection and Food Research Centre, Agriculture and Agri-Food Canada, London, Ontario, Canada; c Dairy and Swine Research Development Centre, Agriculture and Agri-Food Canada, Sherbrooke, Quebec, Canada

## Abstract

The draft genome sequence of *Arcobacter cibarius* strain LMG21996^T^, isolated from chicken carcasses, is reported here. The draft genome consists of 2.2 Mbp, with a 27.12% G+C content. A total of 2,179 protein-coding genes, 46 tRNA genes, and 15 rRNAs have been identified and annotated.

## GENOME ANNOUNCEMENT

*Arcobacter cibarius* strain LMG21996^T^ was first isolated from the skin of broiler carcasses and proposed as the type strain of *A. cibarius* by Houf and coworkers (1). Phylogenetic analysis showed close similarity to *Arcobacter cryaerophilus* (97.5%), *A. butzleri* (96.5%), *A. skirrowii* (96.0%), *A. nitrofigilis* (95.0%), and other closely related *Campylobacter* (88%) and *Helicobacter* (87%) species, based on multiple pairwise alignments of 16S rRNA genes.

The draft genome sequence of strain LMG21996^T^ was obtained to contribute to the Genomic Encyclopedia of Type Strains (2). The draft genome sequence of *A. cibarius* LMG21996^T^ was determined by paired-end sequencing using an Illumina HiSeq 2500 with TrueSeq V3 chemistry at the National Research Council Canada (Saskatoon, Saskatchewan, Canada). A total of 30,303,610 paired-end reads totaling 3,060,664,610 bp (each 101 bp in length) were obtained from 300-bp inserts. Quality checking using FastQC (http://www.bioinformatics.babraham.ac.uk/projects/fastqc/) showed that the reads were of excellent quality; therefore, no further trimming or error correction was required. Initial *de novo* assembly using ABYSS v1.3.6 (3) produced 95 contigs contained in 73 scaffolds, from which scaffolds with lengths of <300 bp were removed. The remaining 47 scaffolds (minimum, 628 bp; maximum, 231,348 bp; *N*_50_, 119,037 bp; total size, 2,198,636 bp; total number of unknown nucleotides [Ns], 959) were used for further analyses. SSPACE v2.0 (4) was applied on the resulting scaffolds to possibly extend and merge them into larger scaffolds based on read-pair information and short overlaps. This process reduced the number of scaffolds to 44 (minimum, 628 bp; maximum, 232,220 bp; *N*_50_, 119,037 bp; total size, 2,201,250 bp; total number of Ns, 968). GapFiller v1.11 (5) was used to close the gaps between the short scaffolds contained within the large 44 scaffolds by replacing the unknown nucleotides (Ns) with true nucleotides based on read-pair information and short overlaps. The final draft genome consists of 2,201,349 bp, containing 13 Ns and consisting of 44 scaffolds (minimum, 628 bp; maximum, 232,245 bp; *N*_50_, 119,037 bp). The G+C content of the draft genome is 27.12%, with an overall estimated coverage of 1,302×.

In addition, Mauve contig mover v2.3.1 (6) was applied to order the draft genome of the *A. cibarius* strain LMG21996^T^ using *A. butzleri* ED-1 (GenBank accession no. NC_017187.1) as a reference genome (7). Automated annotation, performed using the RAST annotation server (8), revealed 2,179 predicted protein-coding sequences, of which 1,585 have assigned functions, 281 have proposed functions, and 313 have been considered to encode hypothetical proteins. The draft genome also contains 61 predicted noncoding RNAs, including 45 tRNAs, 1 pseudo-tRNA, and 15 rRNAs. The numbers of gene copies encoding 16S rRNA, 5S rRNA, and 23S rRNA are 3, 6, and 6, respectively. Various other genome annotation programs delivered results comparable to but different from those of RAST. For example, Glimmer v3.02 (9) using open reading frames (ORFs) as a training set predicted 2,199 genes, whereas RNAmmer v1.2 (10) predicted 14 rRNAs containing 3 copies of 16S rRNA, 5 copies of 5S rRNA, and 6 copies of 23S rRNA genes. However, tRNAscan-SE v1.3.1 (11) predicted 46 tRNA genes and 1 pseudo-tRNA gene.

### Nucleotide sequence accession numbers.

This whole-genome shotgun project has been deposited at DDBJ/EMBL/GenBank under the accession number JABW00000000. The version described in this paper is the first version, JABW01000000.
